# ANGPTL3, Apo CIII, Leptin and Triglycerides Are Elevated in Metastatic Prostate Cancer

**DOI:** 10.3390/cancers18071176

**Published:** 2026-04-07

**Authors:** Gabriel Boulay, Marwan Khodr, Ann-Charlotte Bergeron, Émilie Wong Chong, France-Hélène Joncas, Chloé Castonguay, Karine Robitaille, Hélène Hovington, Vincent Fradet, Alain Bergeron, Frédéric Pouliot, Jonatan Blais, Nabil G. Seidah, Frédéric Calon, Anne Gangloff

**Affiliations:** 1 Faculté de Pharmacie, Université Laval, Québec City, QC G1V 0A6, Canada; gabriel.boulay.1@ulaval.ca (G.B.); frederic.calon@pha.ulaval.ca (F.C.); 2 Axe Oncologie, Centre de Recherche du CHU de Québec–Université Laval, Québec City, QC G1V 4G2, Canadavincent.fradet@fmed.ulaval.ca (V.F.); alain.bergeron@crchudequebec.ulaval.ca (A.B.); frederic.pouliot@crchudequebec.ulaval.ca (F.P.); jonatan.blais.med@ssss.gouv.qc.ca (J.B.); 3 Axe Neurosciences, Centre de Recherche du CHU de Québec–Université Laval, Québec City, QC G1V 4G2, Canada; 4 Faculté de Médecine, Université Laval, Québec City, QC G1V 0A6, Canada; marwan.khodr.1@ulaval.ca; 5 Centre de Recherche sur le Cancer (CRC)–Université Laval, Québec City, QC G1J 0J9, Canada; 6 Unité de Recherche en Biochimie Neuroendocrinienne, Institut de Recherches Cliniques de Montréal, Montréal, QC H2W 1R7, Canada; nabil.seidah@ircm.qc.ca

**Keywords:** ANGPTL3, APOC3, leptin, triglycerides, Lp(a), metastatic prostate cancer, lipid-lowering drugs

## Abstract

Background/Objectives: Prostate cancer (PCa) cells are known to heavily depend on lipids to support their growth. We hypothesized that hyperlipidemic factors, for which inhibitors are already available and used to treat cardiovascular disease, would be dysregulated in metastatic PCa (mPCa). The goal of this case-control study, including 35 men per group, was to compare the levels of PCSK9, ANGPTL3, Apo CIII, leptin, and the lipid profile in patients with mPCa versus localized Gleason 8/9 PCa (lPCa) and patients at risk of developing PCa (controls). Methods: Protein levels were assessed using ELISAs, while lipids were measured using the Roche Cobas analytical platform. Results: The following circulating analytes were higher in mPCa: triglycerides (in mmol/L; controls 1.7 ± 1.2, lPCa 1.5 ± 0.7, mPCa 2.3 ± 1.2, *p* = 0.0004), Apo CIII (in µg/mL; control 110.7 ± 55.7, lPCa 115.0 ± 57.64, mPCa 159.9 ± 96.7, *p* = 0.0179), ANGPTL3 (in ng/mL; controls 41.7 ± 20.0, lPCa 42.8 ± 24.1, mPCa 57.3 ± 26.9, *p* = 0.0390), and leptin (in ng/mL, controls 9.6 ± 9.1, lPCa 8.2 ± 7.9, mPCa 17.7 ± 17.8, *p* < 0.0001). Surprisingly, PCSK9 levels were negatively correlated with LDL in the entire cohort. Conclusions: In this cohort of men, whole-body lipid metabolic rewiring is a feature restricted to the metastatic phase of prostate cancer, suggesting it may play a significant role in the progression toward more aggressive cancer forms. Given the availability of drugs targeting ANGPTL3 and Apo CIII, the therapeutic potential of these drugs should be evaluated in metastatic PCa.

## Simple Summary

Prostate cancer is particularly dependent on lipids to support its growth. Recently, new therapeutic approaches targeting hyperlipidemic factors, which allow a tight control of lipid metabolism, have been successfully employed to manage cardiovascular diseases. Our study aimed to quantify the levels of a number of these proteins, namely PCSK9, ANGPTL3, Apo CIII and leptin, in metastatic prostate cancer patients compared to localized Gleason 8/9 prostate cancer and patients at risk of developing prostate cancer (35 men per group). Whole-body metabolic reprogramming in prostate cancer patients could promote disease progression by providing fuel to cancer cells.

## 1. Introduction

Prostate cancer (PCa) is the most commonly diagnosed cancer and one of the deadliest cancers among men in Canada [1]. Early detection results in over 90% survival rate for 15 years [2]. However, around one-third of patients will experience biochemical recurrence within 5 years of local therapy, and most of these patients will present metastases [3,4]. Androgen pathway inhibitors can block PCa growth but are effective only for a limited time before promoting the differentiation of castration-resistant prostate cancer cells (CRPC). Thus, the median survival for patients with CRPC ranges from 9 to 30 months, which decreases to 9 to 13 months if the cancer becomes metastatic [5].

**Figure 1:**
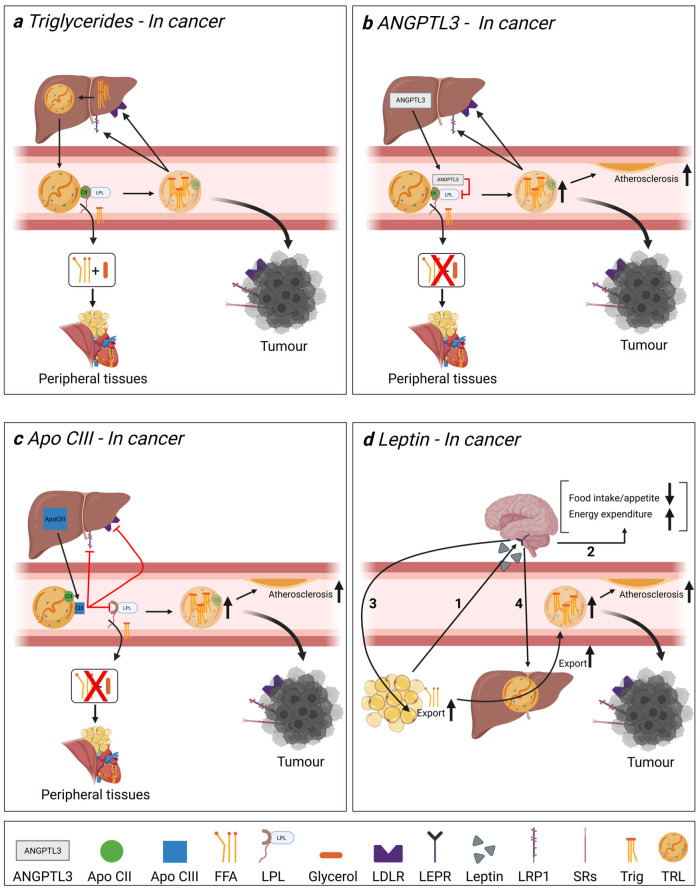
Roles of ANGPTL3, Apo CIII, and leptin in delivering triglycerides to tissues and cancer cells. Similarly to regular cells, cancer cells can obtain lipids from triglyceride-rich lipoproteins (TRLs) originating from dietary lipid uptake transferred into chylomicrons or through very-low-density lipoproteins (VLDLs) originating from the liver. (**a**) Triglycerides are delivered to peripheral tissues (adipose, muscles, and heart) through TRLs for energy use and storage. This process is regulated by the breakdown of triglycerides into fatty acids using lipoprotein lipase (LPL). Apo CII found on lipoproteins activates LPL, allowing the hydrolysis of triglycerides into free fatty acids and their subsequent uptake by tissues. Large TRLs can be transformed into intermediate-density lipoproteins (IDLs), low-density lipoproteins (LDLs), and TRL remnants as their size diminishes. LDL particles interact with LDL receptors (LDLR) present on the surface of the liver and peripheral tissues, where they can be internalized and processed in endosomes, leading to the removal of cholesterol from the blood. (**b**) ANGPTL3 is primarily produced by the liver and can have far-reaching effects, inhibiting hepatic lipase, endothelial lipase, and lipoprotein lipase found in capillaries that supply distant peripheral tissues [29,30,31]. ANGPTL3 activity is regulated in response to feeding status, as has been reviewed by Sylvers-Davie and Davies 2021 [29]. (**c**) Apo CIII is secreted by the liver and, to a lesser extent, by the intestine. Apo CIII reduces the hydrolysis of triglycerides by LPL, thereby reducing fatty acid tissue uptake and leading to higher circulating triglyceride levels. Apo CIII also negatively regulates Apo E-mediated hepatic uptake of TRLs and lipoprotein remnants via the LDL receptor (LDLR) and LDLR-related protein 1 (LRP1) [32,33,34]. (**d**) Leptin is secreted by adipocytes in correlation with the amount of stored fat deposits, aiding in the homeostasis of adipose tissue (1) [35]. It regulates satiety and energy balance by acting on leptin receptors located in the brain, primarily in the hypothalamus, and also acts on peripheral tissues, such as the liver, pancreas, prostate, and adipocytes (2) [36]. Leptin stimulates lipolysis and fatty acid oxidation while simultaneously blocking lipogenesis in adipose tissue [37,38], leading to an efflux of fatty acids from fat stores into the blood (3). Circulating free fatty acids are then processed by the liver to be repackaged into triglycerides and secreted as TRLs. As such, leptin further increases circulating triglyceride levels (4) [39,40]. In summary, ANGPTL3, Apo CIII and leptin can regulate circulating triglyceride levels in the blood, increasing their availability for tumour cells. LDLR, LRP1, and scavenger proteins found on cancer cells mediate TRL uptake and internalization, supplying essential lipids to tumours in great quantity, which supports their growth [41,42]. Apo CII: apolipoprotein CII, Apo CIII: apolipoprotein CIII, FFA: free fatty acids, LDLR: LDL receptor, LEPR: leptin receptor, LPL: lipoprotein lipase, LRP1: low-density lipoprotein receptor-related protein 1, SRs: lipid scavenger receptors, Trig: triglycerides, TRL: triglyceride-rich lipoprotein. Created in BioRender. Boulay. G. (2026) https://BioRender.com/q7mc1m0.

A patient’s risk of metastatic progression may stem from a systemic predisposition that creates a favourable environment for cancer spread. Hyperlipidemia has been linked to cancers, including PCa, and is associated with a heightened risk of aggressive and metastatic disease in numerous cases [5,6,7,8,9]. Although lipids are known to contribute to PCa progression, conflicting data exist regarding changes in lipid profiles in PCa and the precise roles of lipids and lipid-related factors in the progression of the disease. Angiopoietin-like protein 3 (ANGPTL3) [10,11,12,13,14], apolipoprotein CIII (Apo CIII) [10], leptin [15,16,17,18,19,20,21], proprotein convertase subtilisin/kexin type 9 (PCSK9) [11,12,22,23,24,25] and lipoprotein(a) (Lp(a)) [26,27,28] levels have been shown to be altered in several cancers. However, the effect of PCa grade or stage on circulating levels of these proteins remains an open question and a gap that the current study seeks to address. A review of the physiological actions of ANGPTL3, Apo CIII, and leptin on lipid metabolism is provided in Figure 1 to put our work into context.

To support cancer growth, cancer cells acquire receptors that enable them to extract lipoproteins and their lipids from the bloodstream, including cholesterol (a building block for membranes) and free fatty acids (cellular fuel) derived from triglyceride-rich lipoproteins (TRLs) [41,42,43,44]. As such, ANGPTL3, Apo CIII, leptin, and PCSK9 are critical proteins that modulate lipid metabolism and may promote cancer progression. Unfortunately, significant gaps in our understanding of their regulation in patients with prostate cancer, particularly metastatic PCa, persist. Given the availability of inhibitors [31], a new therapeutic avenue could rapidly be developed if one or several of these lipid-related proteins are causally linked to poor PCa prognosis. This study reports, for the first time, the circulating levels of Apo CIII, ANGPTL3, leptin, PCSK9, and Lp(a) in patients with metastatic PCa and compares them to localized Gleason 8 or 9 PCa and controls.

## 2. Patients and Methods

### 2.1. Selection of Plasma Samples

Plasma samples from three distinct groups of participants were analysed. The control group included 35 men at risk of PCa, referred for a first prostate biopsy either following a measurement of elevated prostate specific antigen (PSA) levels (laboratory reference intervals (in ng/mL): 20–29 years: <1.10; 30–39 years: <1.50; 40–49 years: <2.00; 50–59 years: <3.00; 60–69 years: <4.00; >70 years: <6.00) or a positive family history of PCa. These men had negative prostate biopsies and remained cancer-free for at least 3.2 years following biopsy and blood sampling. The localized high-grade prostate cancer (lPCA) group comprised 35 men with a Gleason score of 8 or 9 and treated by radical prostatectomy. Finally, the third group consisted of men with metastatic prostate cancer (mPCa). The 35 mPCa patients with the highest PSA values measured at some point in their treatment history were chosen. No exclusion criteria were applied, provided that the samples were from men who met all inclusion criteria. Selected samples were matched for BMI between groups. Furthermore, correcting for age and BMI did not change the significance of the results. Plasma samples for the cancer-free group were obtained from the Banque d’échantillons biologiques et de données pour la recherche sur les maladies de la prostate (BIOCaPPE) biobank, while samples for the localized and metastatic groups were sourced from the Banque de tissus et de données médicales pour la recherche en urologie au CHU de Québec–Université Laval (URO-1) biobank. Both biobanks are registered with the CHU de Québec-Université Laval hospital (Projects #2012-1111 and #2012-1002, respectively). All participants provided informed consent prior to biosample and data collection.

Blood samples were collected between 2010 and 2021 using vacutainers containing EDTA. Plasma was separated from whole blood by centrifugation and stored at −80 °C. Participants’ data, including sex, age, body mass index (BMI), PSA levels, tumour staging, biochemical recurrence, and metastasis, were provided by the biobanks. The current study, using biosamples and data from the biobanks, was approved by the ethics committee of the CHU de Québec–Université Laval (approval number 2023-6537).

### 2.2. Assessment of Apo B, Lp(a) and the Lipid Profile

Apolipoprotein B100 (Apo B, g/L), Lp(a) (nmol/L), and PSA (ng/mL), along with a detailed lipid profile, were measured using a Roche Cobas^®^ modular analytical platform at the Core Laboratory of Hôpital de l’Enfant-Jésus (Quebec City, QC, Canada). The lipid profile assessment included total cholesterol (TC, mmol/L), HDL cholesterol (HDL, mmol/L), and triglycerides (Trig, mmol/L). LDL cholesterol (LDL, mmol/L) levels were calculated using the Friedewald formula: LDL = TC − (Trig/2.2) − HDL. Non-HDL cholesterol (non-HDL, mmol/L) levels were calculated as TC–HDL. For Lp(a) measurements that fell below the reportable range (<10 nmol/L), a default value of 3 nmol/L, half of the lower limit of detection, was assigned.

### 2.3. Assessment of PCSK9, ANGPTL3, Apo CIII and Leptin Levels by ELISA

Following the manufacturers’ instructions, ANGPTL3, Apo CIII, leptin and PCSK9 levels were measured with enzyme-linked immunosorbent assay (ELISA) kits. ANGPTL3 levels were assessed using the SimpleStep ELISA^®^ Kit (#ab254510, Abcam, Cambridge, MA, USA); Apo CIII levels were evaluated with the Human Apolipoprotein CIII ELISA Kit (#Ab154131, Abcam, Cambridge, MA, USA); leptin levels were determined using the Human Leptin ELISA Kit (#KAC2281, Invitrogen, Camarillo, CA, USA); and PCSK9 was evaluated with the Legend Max^TM^ ELISA Kit (#443107, Biolegend, San Diego, CA, USA). Every sample was measured twice on the same day. Intra-assay coefficient of variability (CV) averaged 5.08% for ANGPTL3, 20.97% for Apo CIII, 11.34% for leptin, and 3.08% for PCSK9. Assay-to-assay variability between ELISA plates (inter-assay CV) was 16.90% for ANGPTL3, 25.54% for Apo CIII, 15.05% for leptin, and 9.22% for PCSK9.

### 2.4. Statistical Analyses

A sample size of 35 men per group ensured a statistical power of 80% and an alpha error of 0.05, as was estimated using G*Power software version 3.1. Statistical analyses were conducted using R software version 4.2.3, JMP Pro version 17.2, and SAS version 9.4. Significance thresholds were defined as follows: * *p* < 0.05, ** *p* < 0.01, *** *p* < 0.001, **** *p* < 0.0001.

A Shapiro–Wilk test was used to assess the Gaussian distribution of the data. Data following a Gaussian distribution underwent an analysis of variance (ANOVA) and an analysis of covariance (ANCOVA) on logarithmically transformed data using age and BMI as covariates. Non-Gaussian parameters were analysed using a Kruskal–Wallis test and a generalized linear model (GLM) with age and BMI as covariates. Using SAS, the ideal GLM distribution (Tweedie) was ascertained for ANGPTL3, leptin and Lp(a). Group differences were assessed by two-tailed post hoc pairwise comparisons for variables with statistically significant *p*-values (*p* < 0.05) using a Tukey–Kramer adjustment for multiple comparisons. GraphPad Prism version 8.0.2 was used to visualize the data in boxplots or scatterplots. Partial Spearman correlations (rho coefficients) and their *p*-value (t-distribution approximation with *n* − 2 degrees freedom) were calculated using the Corx package version 1.0.7.2 (courtesy of Dr. James Conigrave, https://github.com/conig/corx) and R version 4.2.3 to assess the associations between variables in the entire cohort and for each subgroup (at risk, lPCa and mPCa), admitting a two-tailed hypothesis. Furthermore, a multiple regression was conducted for triglycerides to evaluate their correlation to statistically significant variables found in the preceding analysis.

## 3. Results

### 3.1. Study Population Characteristics

Diagnosis. The biological characteristics of patients at risk of PCa (controls, *n* = 35), patients diagnosed with locally advanced Gleason 8 and 9 PCa (*n* = 35), and patients diagnosed with metastatic PCa (*n* = 35) are listed in Table 1. Blood samples were collected from fasting men in the localized and at-risk groups, except for one non-fasting participant in each. However, the fasting status of the metastatic group is unknown.

Age. The mean age for this cohort of men was 69 years old (68.9 ± 8.4 y. o.). Age did not differ between men at risk of PCa vs. men with lPCa, but males with mPCa were significantly older (at risk 66.7 ± 7.3, lPCa 66.7 ± 7.4, mPCa 73.4 ± 8.7 y. o., *p*-value = 0.0006; Table 1), a trend that was expected given that age is a strong risk factor for metastasis.

BMI. No significant difference in BMI was observed between the three groups (in kg/m^2^; at risk 28.5 ± 3.8, lPCa 28.7 ± 4.1, mPCa 27.5 ± 4.1, *p*-value = 0.197). All groups of men were overweight, as defined by a BMI of 25.0 to 29.9 kg/m^2^ (Table 1).

Treatment. Men at risk of PCa did not receive any PCa treatment. Eight patients in the localized group received a 5-alpha reductase inhibitor, although one patient had stopped receiving treatment at the time of sample collection. All patients in the metastatic group received a combination of luteinizing hormone-releasing hormone (LHRH) agonists and antiandrogens, except for two patients who received only an LHRH agonist. One patient underwent surgical castration prior to sample collection (Table 1).

### 3.2. Lipid Profile in Controls and Localized PCa Versus Metastatic PCa

**Figure 2:**
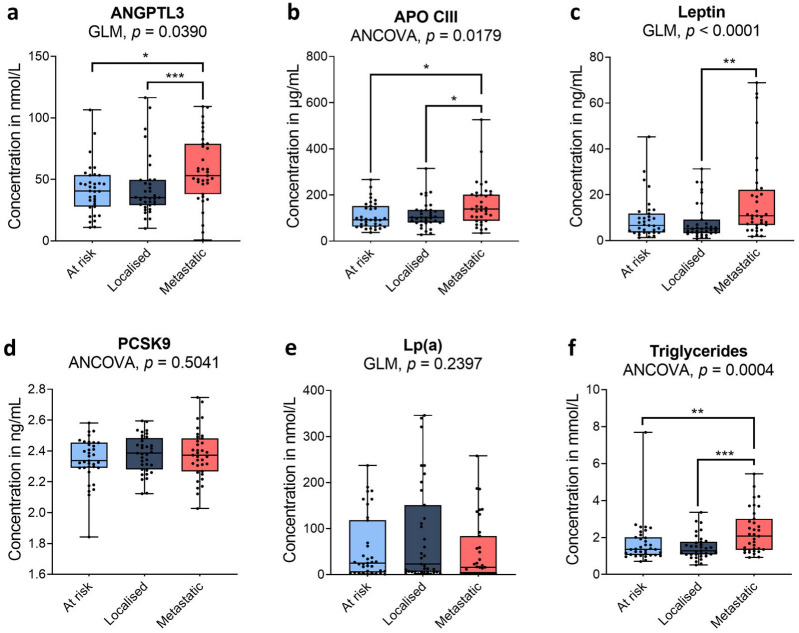
Box plots showing circulating levels of ANGPTL3 (**a**), Apo CIII (**b**), leptin (**c**), PCSK9 (**d**), Lp(a) (**e**), and triglycerides (**f**) between men at risk of PCa (controls), men with localized high-grade PCa and men with metastatic PCa (*n* = 35/group). The centre line represents the median, the box spans the 1st and 3rd quartiles, and the whiskers indicate the smallest or largest values. A two-sided ANCOVA using age and BMI as covariates was performed on log-transformed data of variables that showed a Gaussian distribution, and a two-sided generalized linear model (GLM) using the Tweedie distribution with age and BMI as covariates was applied to variables with non-Gaussian distributions. *: *p* < 0.05, **: *p* < 0.01, ***: *p* < 0.001.

Triglycerides were markedly increased in mPCa, a finding not observed in lPCa, when compared to patients at risk of PCa (in mmol/L; controls 1.7 ± 1.2, lPCa 1.5 ± 0.7 and mPCa 2.3 ± 1.2, *p*-value = 0.0004; Figure 2 and Table 1). Other than triglycerides, the lipid profile remained unchanged in men at risk of PCa, those with localized PCa or those with metastatic PCa. Levels of total cholesterol, HDL, non-HDL, LDL and Apo B were comparable among the three groups, as seen in Table 1. Lp(a) levels did not change significantly among groups of men (in nmol/L; controls 57.5 ± 69.1, lPCa 85.1 ± 106.4, mPCa 49.7 ± 66.4).

### 3.3. Lipid-Related Factors in Controls and Localized PCa Versus Metastatic PCa

Lipid-related factors were significantly altered in metastatic PCa, even after adjusting for age and BMI.

**Figure 3:**
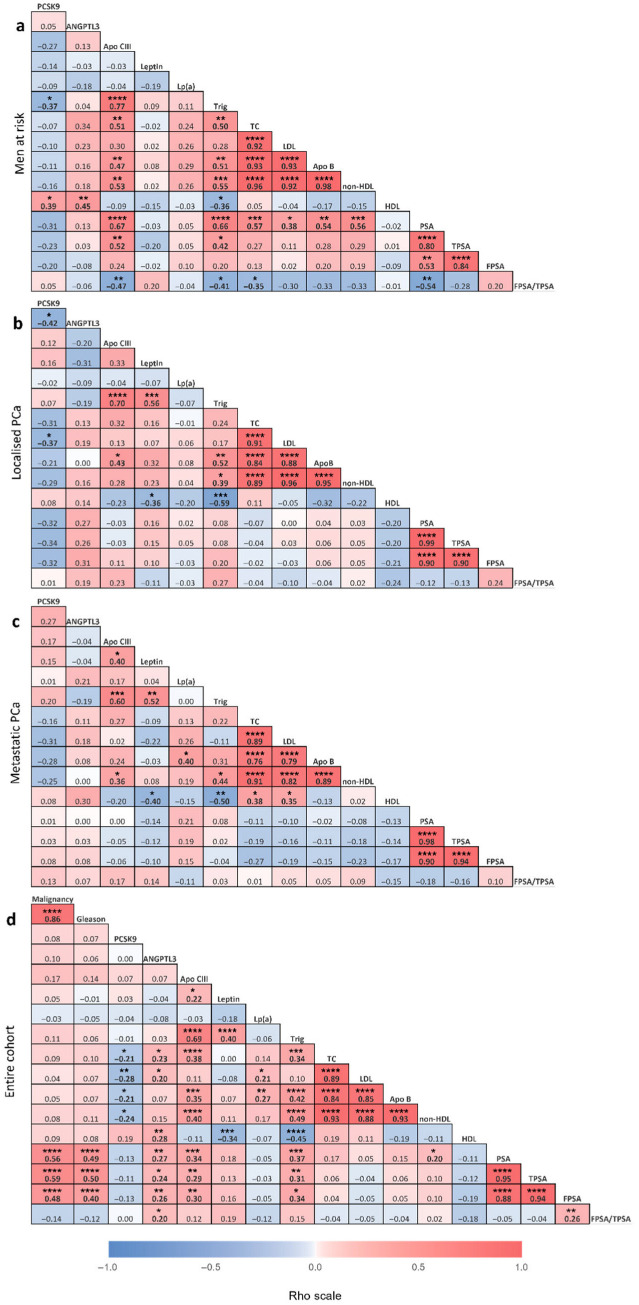
Matrices of pairwise Spearman’s correlation analyses between all study variables. Pairwise correlations are shown for patients at risk of PCa ((**a**), *n* = 35), patients diagnosed with localized high-grade PCa ((**b**), n = 35), patients diagnosed with metastatic PCa ((**c**), *n* = 35), and the entire cohort ((**d**), *n* = 105). Correlation coefficients (rho) are displayed with colour hues indicative of relationship strength (red: positive correlation, blue: negative correlation). Significant values are indicated in boldface (two-sided *p*-values: *: *p* < 0.05, **: *p* < 0.01, ***: *p* < 0.001, ****: *p* < 0.0001).

The concentration of ANGPTL3 increased in metastatic PCa patients, while it did not change in the lPCa group (in ng/mL: control group 41.7 ± 20.0, lPCa 42.8 ± 24.1 and mPCa 57.3 ± 26.9, *p*-value = 0.0390; Figure 2). Except for HDL in the control group, ANGPTL3 did not show a significant correlation with any other analyte when each group was analyzed separately (Figure 3a–c). However, ANGPTL3 was significantly correlated with total cholesterol, LDL, and HDL in the entire cohort (Figure 3d).

Apo CIII levels increased in metastatic PCa patients (in µg/mL; controls 110.7 ± 55.7, lPCa 115.0 ± 57.6 and mPCa 159.9 ± 96.7 µg/mL, *p*-value = 0.0179; Figure 2 and Table 1). Apo CIII was positively associated with triglycerides in each subgroup (Figure 3a–c). In the entire cohort (Figure 3d), the positive associations between Apo CIII and the tumour markers (PSA, TPSA, FPSA) and between Apo CIII and atherogenic lipids (total cholesterol, triglycerides, Apo B, and non-HDL) became significant.

The circulating concentration of leptin also increased in metastatic cancer (in ng/mL; controls 9.6 ± 9.1, lPCa 8.2 ± 7.9, mPCa 17.7 ± 17.8, *p*-value < 0.0001; Figure 2). In the entire cohort of 105 men, leptin associated positively with triglycerides and Apo CIII and negatively with HDL.

**Figure 4:**
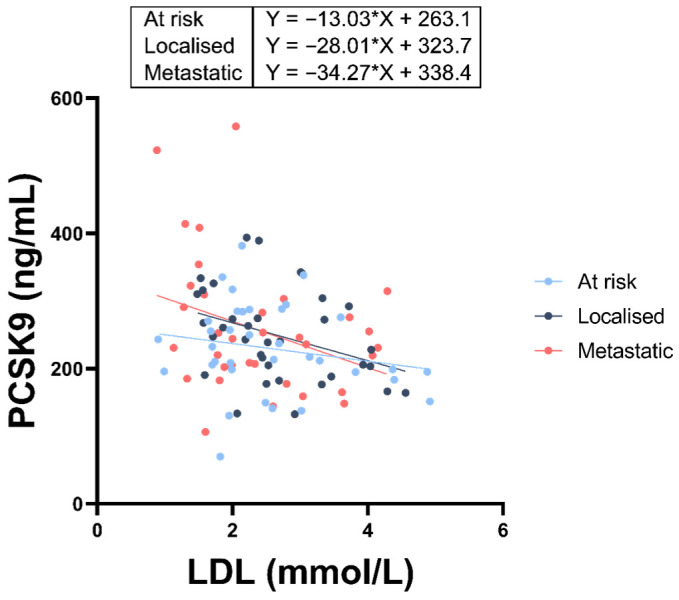
PCSK9 levels as a function of circulating LDL. A linear regression shows an inverse correlation between PCSK9 and LDL levels in men at risk of prostate cancer (PCa), men with localized PCa and men with metastatic PCa (*n* = 35/group). This finding is unexpected as PCSK9 elevates cholesterol by destroying hepatic LDL-R. The slope of the linear regression is shown on the graph.

PCSK9 levels did not vary between the three groups (Figure 2). Few associations were found between PCSK9 and other analytes within a given group (Figure 3a–c). Notably, in the entire cohort, once adjusted for age and BMI, PCSK9 exhibited a *negative* correlation with atherogenic lipoproteins, including total cholesterol (ρ = −0.21, *p* = 0.0363), LDL (ρ = −0.28, *p* = 0.0039), non-HDL (ρ = −0.24, *p* = 0.0166), and Apo B (ρ = −0.21, *p* = 0.0322; Figure 3d and Figure 4).

### 3.4. Verifying Known Associations Within the Lipid Profile (Validation Tool)

We performed Spearman correlation analyses to investigate if localized and metastatic PCa modifies the associations between the lipids and lipid-related factors. Associations between lipid factors, age, and BMI have been documented, such as increased leptin with higher BMI. Accordingly, the analyses were further adjusted for these two factors (age and BMI). The complete matrices of pairwise Spearman’s correlation analyses between all study variables, for each group and in the entire cohort, are presented in Figure 3. Given the high number of variables studied, only the most relevant ones that were part of our initial statistical plan are described below.

Analytes reflecting atherogenic particles (total cholesterol, Apo B, non-HDL and LDL) were strongly associated together across all groups, as expected, with rho coefficients > 0.76 and *p*-values < 0.0001 (Figure 3). Analytes reflecting triglyceride-rich lipoproteins demonstrated robust associations among themselves, specifically between triglycerides, Apo B, and non-HDL, in all subgroups and the entire cohort. The well-established inverse correlation between HDL and triglycerides [45] was observed in our cohort and was even stronger in lPCa (ρ = −0.59, *p* = 0.0003; Figure 3b) and mPCa (ρ = −0.50, *p* = 0.0033; Figure 3c) than in the control group (ρ = −0.36, *p* = 0.0409; Figure 3a). The association between total cholesterol and triglycerides exists in the control group and in the entire cohort (controls ρ = 0.50, *p* = 0.0033; entire cohort ρ = 0.34, *p* = 0.0004; Figure 3a,d) but fails to reach statistical significance in lPCa (ρ = 0.24, *p* = 0.1865; Figure 3b) and mPCa (ρ = 0.22, *p* = 0.2187; Figure 3c).

Interestingly, triglycerides are associated with Apo CIII and leptin in the cancer groups and in the entire cohort (Figure 3b–d). Multiple regression analysis of triglycerides and the hyperlipidemic factors shows that Apo CIII (*p* < 0.0001) and leptin (only in lPCa *p* = 0.0002) predict triglyceride levels (Table A1).

### 3.5. Associations with Tumour Markers

Two measurements of PSA were recorded: the first measured by the hospital at the time of individual sample collection from each patient (referred to as PSA herein), and the second measured in batch from stored frozen samples obtained from the biobank at the time of lipid profiling (referred to as TPSA herein). PSA measurements were recorded using the same analytical platform (Roche Cobas) in both instances.

PSA levels were higher in men with PCa, especially in patients with metastatic PCa, which is an expected finding (in ng/mL; controls 5.8 ± 4.7, lPCa 13.0 ± 12.2, mPCa 174.1 ± 345.6, *p*-value < 0.0001; Table 1). High inter-patient variability was observed in the mPCa group, as indicated by the large standard deviation (SD) value of 345.6 ng/mL for PSA in mPCa, which is likely due to our selection of mPCa patients with the highest PSA values. Although TPSA similarly increased with disease progression (in ng/mL; controls 5.4 ± 3.1, lPCa 15.6 ± 14.9, mPCa 214.0 ± 395.4; Table 1), measurements were slightly different, which had an impact on Spearman correlation analyses shown below. Free PSA levels also increased in the PCa groups, especially in the metastatic group, compared to controls (in ng/mL; controls 0.78 ± 0.5, lPCa 1.7 ± 1.9, mPCa 27.7 ± 34.2, *p*-value < 0.0001; Table 1). Finally, the free/total PSA ratio significantly increased in the mPCa group (controls 0.15 ± 0.06, lPCa 0.12 ± 0.06, mPCa 0.20 ± 0.11, *p*-value = 0.0006; Table 1).

As such, PSA, TPSA, and FPSA were associated with malignancy and Gleason score, as expected (Figure 3d). While PSA measured on fresh samples correlated strongly with atherogenic analytes from the lipid profile in the control group (Figure 3a), using the TPSA measurement from frozen samples weakened those associations (Figure 3a). When the entire cohort is considered (*n* = 105), both PSA and TPSA measurements correlate with many lipid and lipid-related factors such as ANGPTL3, Apo CIII and triglycerides (associations with TPSA: ANGPTL3 ρ = 0.24, *p* = 0.0168; Apo CIII ρ = 0.29, *p* = 0.0031; triglycerides ρ = 0.31, *p* = 0.0018; Figure 3d). Only non-HDL differs between PSA and TPSA (ρ = 0.20, *p* = 0.0418 and ρ = 0.10, *p* = 0.3321, respectively). Free PSA was not measured on fresh samples; instead, it was assessed on samples that had been frozen. The association between free PSA and ANGPTL3 or Apo CIII reached statistical significance only in the overall cohort of patients (ANGTPL3 ρ = 0.26, *p* =0.0087, Apo CIII ρ = 0.30, *p* = 0.0022; Figure 3d).

## 4. Discussion

Prostate cancer cells rely on a steady supply of lipids. Metastatic and castration-resistant PCa cells display activation of fatty acid and cholesterol import and biogenesis pathways [41,42,46]. Contrary to most cancer cells, PCa cells favour fatty acid import over glucose intake [47]. The findings of the present study indicate a correlation between metastatic PCa and altered whole-body lipid metabolism. The current work identified increases in ANGPTL3, Apo CIII, and leptin, all of which contribute to higher levels of triglyceride-rich lipoproteins (TRLs), as reflected by elevations in triglycerides, from which metastatic PCa cells can obtain energy to fuel their growth. We can thus hypothesize that the metastatic status triggers changes in the lipid supply pathways. In light of the evidence presented here, disrupting lipid acquisition by PCa cells emerges as a compelling therapeutic avenue.

### 4.1. Triglycerides in Metastatic PCa

The current study demonstrates significantly higher triglyceride levels in mPCa compared to lPCa and controls (Table 1). Elevated triglyceride levels are thus associated with high PSA levels (Figure 3), as expected for a more advanced disease. An increased risk of aggressive PCa [48] and recurrence [49] has been reported with higher triglyceride levels. Taken together, these results suggest that triglycerides may promote cancer progression and metastasis by supplying energy to rapidly growing cancer cells. Therefore, regulating triglyceride levels could be a promising therapeutic approach, especially given the availability of numerous drugs targeting triglycerides.

### 4.2. Hyperlipidemic Factors

While 20% of the variation in plasma triglycerides can be explained by hepatic production of triglyceride-rich very-low-density lipoproteins (VLDLs), impaired removal of triglyceride-rich lipoproteins accounts for about 55% of the variation in plasma triglyceride levels [32]. Increased circulating levels of ANGPTL3, Apo CIII and leptin were observed in mPCa but not lPCa compared to controls (Figure 2). Apo CIII and ANGPTL3 regulate triglyceride clearance by inhibiting its hydrolysis by LPL [31,32,33] and regulating its uptake by the LDL receptor (LDLR) and LDLR-related protein 1 (LRP1) [29,34,50], whereas leptin primarily induces fatty acid liberation from adipocytes and VLDL secretion from the liver [38]. These pathways could work together to contribute to the high triglyceride levels observed in mPCa. This is supported by our correlation analyses showing that triglycerides are strongly associated with Apo CIII under all conditions and with leptin uniquely in patients with PCa (Figure 3), indicating that a shift in leptin regulation of triglycerides may contribute to increased triglycerides in the blood of PCa patients. Alteration of the leptin–triglyceride association seems to occur before cancer metastasis and subsequent leptin increase (Figure 2 and Figure 3a,b, respectively). Multiple regression analysis of triglycerides and the hyperlipidemic factors indicates that Apo CIII (*p* < 0.0001) and leptin (only in lPCa *p* = 0.0002) predict triglyceride levels (Supplementary Table S1), suggesting that they are key mediators of altered lipid metabolism in PCa. These results suggest that increased triglyceride production induced by leptin could exacerbate circulating triglyceride accumulation caused by Apo CIII inhibition of LPL. Lipid rewiring, as observed here, could effectively divert triglycerides from peripheral tissues to the primary tumour and metastases. Although ANGPTL3 does not correlate with triglycerides, it is associated, alongside Apo CIII, with PSA.

ANGPTL3, Apo CIII, leptin, and PCSK9 are best known for their roles in lipid metabolism. However, these proteins also mediate important functions that could directly affect cancer cells. ANGPTL3, Apo CIII, and leptin promote angiogenesis, atherosclerosis and inflammation [51,52,53]. Furthermore, leptin is a master regulator of countless pathways involved in carcinogenesis, notably affecting cell proliferation, apoptosis, epithelial–mesenchymal transformation, invasion, immune evasion and metastasis [20,21,54]. As for PCSK9, it has been shown to destroy MHC-1, which presents antigens to the immune system. As such, PCSK9 could promote immune escape of tumours, while a PCSK9 inhibitor would preserve immune recognition by inhibiting PCSK9’s action on MHC-1 [23,24,25,55]. Furthermore, PCSK9 also enhances the degradation of CD36, which acts as a signalling receptor and a transporter of long-chain fatty acids (LCFAs), playing a critical part in lipid metabolism, immune responses, and inflammation by clearing LCFAs and removing approximately 50% of oxidized low-density lipoprotein (ox-LDL) from plasma [56,57].

### 4.3. LPL: The Target of ANGPTL3 and Apo CIII

Elevation of Apo CIII, ANGPTL3, and triglycerides all point towards the activity of a common enzyme: lipoprotein lipase (LPL). LPL meets local energetic demands by catalysing triglyceride hydrolysis from TRLs into free fatty acids, a cellular fuel. LPL is the rate-limiting enzyme for triglyceride uptake into tissues [29,58,59]. It is bound to the endothelial cell surface through an interaction with heparin sulphate proteoglycans. Measuring LPL activity is uncommon in clinical practice as it comes with several challenges. Heparin must be administered to make LPL free in the blood, and thus measurable, a procedure with a bleeding risk. As such, LPL activity was not measured in the current study. A paper from 1972 [60] showed that LPL response to heparin (and thus its activity) is decreased in PCa patients compared to controls.

LPL is an extracellular enzyme, expressed in smooth muscles, skeletal muscles and adipose tissues [58,61]. The role of LPL in cancer is complex. It is possible that increasing ANGPTL3 and Apo CIII concentrations inhibits LPL in the whole body, thereby favouring PCa by increasing triglycerides in the blood and diverting TRLs from peripheral tissues toward cancer cells, which could uptake the entire TRL instead of simply hydrolyzing its triglyceride content. From an oncological standpoint, it seems counterintuitive that a tumour would inhibit LPL activity, as this could limit triglyceride hydrolysis and fatty acid uptake by tumour cells. However, LPL is scarcely expressed in PCa cells [62]. As such, prostate cancer cells could obtain all the free fatty acids they need through other mechanisms, possibly via Apo E-mediated uptake of TRLs [63] or directly from free fatty acids carried by albumin and other proteins [64].

Interestingly, Narita et al. have described a gene polymorphism (S447X) leading to increased LPL enzymatic activity [65]. This polymorphism was associated with an increased risk of PCa, especially high-grade and/or stage, in the Japanese population, suggesting that LPL expression by cancer cells could be advantageous to them. Genetic and epigenetic inactivation of the LPL gene in human PCa tissues has been reported by Kim et al. [66]. While LPL is downregulated by promoter hypermethylation in primary prostate tumours, LPL expression increases in metastatic tumours, suggesting a role in tumour progression and metastasis and a possible compensatory mechanism for whole-body LPL inhibition in the cancer microenvironment. These results suggest that a complex equilibrium in triglyceride metabolism influences PCa progression. It also suggests that local expression of LPL versus whole-body expression needs to be considered when assessing the directionality of triglyceride hydrolysis and fatty acid delivery.

### 4.4. Lipoproteins and Cholesterol

In our study, levels of total cholesterol, LDL cholesterol, HDL cholesterol and non-HDL cholesterol were similar between groups (Table 1). Although a Mendelian randomization trial predicted higher Lp(a) levels in men with advanced PCa (defined as metastatic, Gleason score ≥ 8 or PSA > 100 ng/mL, or PCa death) compared to controls [26], our results suggest no association between Lp(a) and metastatic PCa. Other studies have reported conflicting findings with regard to Lp(a) levels in PCa [27,28]. Since our cohort was small, we cannot exclude that differences exist that were not identified here.

Although no changes were observed in circulating cholesterol levels, changes in cholesterol turnover cannot be excluded. PCa cells have the ability to recuperate a greater amount of cholesterol from the blood through a shift in cholesterol import via LDLR and scavenger receptors, accumulating esterified cholesterol in lipid droplets within cells [41,42]. This change would allow PCa cells to synthesize androgens and generate cell membranes, driving tumoural proliferation, signalling and metastatic transformation. In this sense, numerous studies support a role for cholesterol in PCa progression and recurrence [67].

### 4.5. PCSK9 and PCa

PCSK9 levels were similar in all groups (Figure 2). PCSK9 activity can lead to hypercholesterolemia by raising circulating levels of LDL [68,69]. It does so by promoting LDLR, VLDLR, LRP1 and apolipoprotein E receptor 2 (Apo ER2) degradation in the liver [70], thereby impairing hepatic clearance of LDL from the bloodstream. This provides the rationale behind administering PCSK9 inhibitors to patients with hypercholesterolemia; by inhibiting PCSK9, LDLR and other hepatic receptors are protected from destruction, allowing them to internalize LDL and leading to lower cholesterol levels. Unexpectedly, a *negative* correlation was observed between PCSK9 and atherogenic particles, including LDL, Apo B, non-HDL and total cholesterol in the entire cohort (Figure 3d). This negative association was driven by the strong correlation found in lPCa and mPCa (Figure 3b,c and Figure 4).

Two hypotheses can be raised to explain this apparently discordant finding. First, ELISA kits measure total PCSK9, not bioactive PCSK9. Phosphorylated PCSK9, which can only be assessed by mass spectrometry, a measure we did not have access to, exhibits higher activity against the LDLR [71]. A change in its state of phosphorylation in mPCa could greatly alter its potency. Second, tumoural uptake of LDL cholesterol may lead to an increased release of PCSK9, thereby maintaining blood cholesterol homeostasis. Other teams have reported an inverse relationship between PCSK9 and LDL in certain instances of dyslipidemia [72,73,74]. A highly dysregulated PCSK9–LDLR ratio has been reported in metastatic CRPC cells (CWR-R1) [5], suggesting that PCa cells can recuperate circulating cholesterol to promote their growth. Lower PCSK9 activity seems to be protective against PCa. Ference et al. [75] found strong epidemiological evidence supporting a reduction in PCa risk from PCSK9 inhibition. Another study showed that PCSK9 inhibition reduced circulating cholesterol levels as well as recurrence and metastasis in a xenograft PCa mouse model [5]. Double-knockout mutations of PCSK9 have been reported in humans, leading to an eight-fold decrease in circulating LDL cholesterol, while the cardiovascular risk in these individuals drops by 88%, with no associated adverse effects [76]. Further research is required to better understand the regulatory mechanisms connecting PCSK9, LDL cholesterol, and prostate cancer progression.

### 4.6. Androgen-Deprivation Therapy

PCa growth is dependent upon androgen receptor signalling. Androgen deprivation therapy (ADT) is critical for the treatment of metastatic PCa, but tumours can acquire the ability to progress without androgen signalling, a stage called CRPC [77]. Furthermore, ADT heightens the risk of cardiovascular complications and diabetes [20,78]. ADT can significantly alter the lipid profile, leading to large increases in total cholesterol (up to 10%), triglycerides (up to 25%) and HDL (8–20%), which may promote CRPC and metastasis [78,79,80,81].

Thus, the use of ADT to treat all mPCa patients is an important confounding factor in our study, especially regarding increased triglyceride levels. However, we did not observe any effect on cholesterol in our cohort, and the effect of ADT on ANGPTL3, Apo CIII, PCSK9, and leptin has scarcely been studied. ANGPTL3 elevation is most probably not due to ADT since increases in ANGPTL3 protein in the blood and in ANGPTL3 mRNA in cancer tissues have already been reported in high-grade ovarian cancer [13,82]. Ovarian cancers are not treated with antiandrogens but rather with carboplatin. Nevertheless, regardless of its cause (due to tumour metastasis or treatment), elevated lipids and hyperlipidemic factors increase cardiovascular risk and provide fuel to cancer cells. Proper management of lipid metabolism in prostate cancer patients is crucial to limit disease progression.

### 4.7. Available Lipid-Lowering Drugs Targeting Triglycerides, Cholesterol and Other Pathways

We have demonstrated that triglycerides are elevated in PCa, and other studies have shown that this increase is linked to both aggressive [48] and recurrent PCa [49]. As such, triglycerides become an interesting target in mPCa. Potent drugs that target triglycerides and fatty acids, ANGPTL3 (evinacumab, Aro-ANG3), Apo CIII (volanesorsen, olezarsen, plozasiran), and PCSK9 (evolocumab) are already used to treat hyperlipidemia and could rapidly be repositioned in prostate cancer treatment. Lipid-lowering drugs have been proven safe and effective and have been used in clinics for years to treat cardiovascular patients, some of them with cancers, with no safety issues.

### 4.8. Limitations

The sample size of 35 is relatively small and might explain the large standard deviations observed in the current study. Substantial intra- and inter-plate variability was observed for the ELISA assays (PCSK9, ANGPTL3, Apo CIII, and leptin), with mean variability exceeding 25% for Apo CIII. The final measured concentrations remain relatively close between groups while still reaching statistical significance. These important variations might introduce a bias in the analyses.

We mitigated the effect of body weight by matching for BMI when selecting men belonging to the at-risk, localized PCa, and metastatic groups. Nevertheless, BMI has several limitations as a surrogate for body composition. BMI does not accurately reflect adipose tissue content, fat distribution, or lean mass. In metastatic prostate cancer, BMI is particularly misleading due to frequent changes in body composition, including sarcopenia and sarcopenic obesity, especially under androgen deprivation therapy. Consequently, reliance on BMI alone limits interpretation of biomarkers that are tightly linked to adipose tissue biology and metabolic state. CT data quantifying the amount of visceral adipose tissue at the L3-L4 level and an assessment of intramuscular adipose tissues, along muscle mass at the level of the thigh, would have been useful information to stratify and adjust for the impact of real visceral adipose tissue volume and sarcopenia. These data were not available in this cohort.

The present results in metastatic patients reflect men seen in the clinic who are treated with ADT, which modifies lipid metabolism, with varying degrees of metastatic burden (Table A2), sarcopenia and changes in body composition. Information about the fasting status of the metastatic group was also not available. The present results cannot support a mechanistic, bias-free explanation of metabolic changes specifically induced by cancer versus sarcopenia or adiposity. As such, the present results should be seen as a description of the combined effects of all factors on ANGPTL3, Apo CIII, leptin and triglycerides, which all increase significantly in this heterogenous group of metastatic men as compared to men with localized cancer or absence of cancer.

## 5. Conclusions

We report a shift in systemic lipid metabolism in metastatic PCa as compared to non-metastatic stages of the disease. Although Lp(a) and PCSK9 levels were not correlated with disease severity, PCSK9 exhibited an inverse relationship with LDL in metastatic disease. This is quite an astonishing finding, hypothesized to be due to a lower bioactive fraction or a compensatory mechanism triggered by peripheral LDL uptake. We observed increased circulating levels of triglycerides, along with rises in factors known to promote higher triglyceride levels, namely ANGPTL3 and Apo CIII. Leptin also contributes to the increased secretion of triglyceride-rich lipoproteins, which was more pronounced in the metastatic stage. Overall, our results demonstrate that whole-body lipid metabolism is altered in metastatic prostate cancer patients treated with ADT and raise the question of whether lipid-lowering treatments should be employed to slow PCa progression and manage cardiovascular risk in these patients.

## Figures and Tables

**Table 1:** Characteristics of the study cohort.

			Prostate Cancer			
	Total (*N* = 105)	At Risk (*n* = 35)	Localized (*n* = 35)	Metastatic (*n* = 35)	*p*-Value	Adjusted *p*-Value
**Anthropometry**						
Age (years)						
Mean (SD)	68.9 (8.4)	66.7 (7.3)	66.7 (7.4)	73.4 (8.7)		
Median	71	67	67	75	**0.0006 ** ^&^	-
[Min, Max]	[50, 90]	[50, 77]	[50, 79]	[58, 90]		
BMI (kg/m^2^)						
Mean (SD)	28.26 (3.98)	28.54 (3.72)	28.71 (4.02)	27.54 (4.08)		
Median	28.17	28.26	28.52	26.70	0.1971 ^&^	-
[Min, Max]	[20.10, 40.98]	[21.44, 40.61]	[20.69, 40.98]	[20.10, 39.00]		
**Grade**						
Gleason Score	*N* = 105	*n* = 35	*n* = 35	*n* = 35		
NA:	35	35	-	-	-	-
6:	1	-	-	1		
7:	14	-	-	14		
8:	33	-	26	7		
9:	21	-	9	12		
10:	1	-	-	1		
**Treatments**						
LHRH agonist/antiandrogen	35	0	0	35	-	-
5-alpha reductase inhibitor	10	0	7	3	-	-
**Tumour Markers**						
PSA (ng/mL) ^1^						
Mean (SD)	64.3 (214.3)	5.8 (4.7)	13.0 (12.2)	174.1 (345.6)		
Median	9.8	5.4	8.4	52.4	**<0.0001 ^=^**	<0.0001 *
[Min, Max]	[0.2, 1931.0]	[0.2, 28.0]	[0.3, 56.0]	[10.3, 1931.0]		
Total PSA (ng/mL)						
Mean (SD)	78.3 (247.8)	5.4 (3.1)	15.6 (14.9)	214.0 (395.4)		
Median	10.9	4.9	9.8	63.1	**<0.0001 ^=^**	<0.0001 *
[Min, Max]	[0.2, 2196.4]	[0.2, 14.1]	[0.4, 65.9]	[1.4, 2196.4]		
Free PSA (ng/mL)						
Mean (SD)	10.1 (23.4)	0.8 (0.5)	1.7 (1.9)	27.7 (34.2)		
Median	1.3	0.6	1.0	16.8	**<0.0001 ^=^**	<0.0001 *
[Min, Max]	[0.1, 171.7]	[0.1, 2.6]	[0.1, 7.7]	[0.2, 171.7]		
FPSA/TPSA						
Mean (SD)	0.16 (0.09)	0.15 (0.06)	0.12 (0.06)	0.20 (0.11)		
Median	0.13	0.14	0.09	0.19	**<0.0001 ^=^**	0.0006 *
[Min, Max]	[0.04, 0.57]	[0.07, 0.31]	[0.04, 0.28]	[0.04, 0.57]		
**Lipid Profile**						
Total Cholesterol (mmol/L)						
Mean (SD)	4.61 (1.07)	4.51 (1.16)	4.64 (0.93)	4.68 (1.10)		
Median	4.46	4.32	4.58	4.51	0.6896 ^=^	0.4777 *
[Min, Max]	[2.49, 7.16]	[2.49, 7.16]	[3.31, 6.48]	[2.95, 6.58]		
HDL (mmol/L)						
Mean (SD)	1.24 (0.34)	1.19 (0.28)	1.31 (0.36)	1.24 (0.35)		
Median	1.18	1.13	1.22	1.14	0.2772 ^=^	0.1952 *
[Min, Max]	[0.68, 2.89]	[0.75, 1.90]	[0.83, 2.89]	[0.68, 2.14]		
Triglycerides (mmol/L)						
Mean (SD)	1.83 (1.09)	1.70 (1.17)	1.47 (0.65)	2.32 (1.17)		
Median	1.46	1.36	1.28	2.07	**0.0006 ^=^**	0.0004 *
[Min, Max]	[0.51, 7.69]	[0.70, 7.69]	[0.51, 3.36]	[0.92, 5.44]		
LDL (mmol/L)						
Mean (SD)	2.54 (0.94)	2.55 (0.99)	2.67 (0.85)	2.40 (0.96)		
Median	2.39	2.25	2.50	2.25	0.3299 ^=^	0.6230 *
[Min, Max]	[0.88, 4.92]	[0.90, 4.92]	[1.48, 4.56]	[0.88, 4.29]		
Apolipoprotein B (g/L)						
Mean (SD)	0.86 (0.25)	0.86 (0.28)	0.87 (0.22)	0.84 (0.24)		
Median	0.79	0.79	0.810	0.77	0.8096 ^=^	0.8739 *
[Min, Max]	[0.37, 1.53]	[0.37, 1.53]	[0.50, 1.40]	[0.50, 1.39]		
non-HDL cholesterol (mmol/L)						
Mean (SD)	3.37 (1.08)	3.32 (1.21)	3.33 (0.97)	3.45 (1.04)		
Median	3.15	3.08	3.14	3.38	0.7580 ^=^	0.3857 *
[Min, Max]	[1.25, 6.29]	[1.25, 6.29]	[1.82, 5.46]	[2.00, 5.54]		
Lipoprotein (a) (nmol/L)						
Mean (SD)	64.09 (84.05)	57.47 (69.08)	85.07 (106.44)	49.74 (66.35)		
Median	23.00	25.00	23.00	16.00	0.3243 ^&^	0.2397 ″
[Min, Max]	[1.20, 346.00]	[1.20, 237.00]	[2.00, 346.00]	[3.00, 258.00]		

SD: Standard deviation, Min: Minimum value, Max: Maximum value. ^1^ PSA measured by the clinic at the time of data collection, before samples were frozen. ^=^ Two-sided ANOVA performed on log-transformed data. ^&^ Two-sided Kruskal–Wallis performed on log-transformed data. * Two-sided ANCOVA performed on log-transformed data with age and BMI as covariates. ″ Two-sided generalized linear model (GLM) using Tweedie distribution with age and BMI as covariates.

## Data Availability

The original contributions presented in this study are included in the article/Supplementary Materials. Further inquiries can be directed to the corresponding author.

## Supplementary Materials

The following supporting information can be downloaded at: https://www.mdpi.com/article/10.3390/cancers18071176/s1, Table S1: Raw_quantification_lipid_factors.

## Abbreviations

The following abbreviations are used in this manuscript:

ANCOVA	analysis of covariance
ANOVA	analysis of variance
ADT	androgen deprivation therapy
ANGPTL3	angiopoietin-like protein 3
Apo B	apolipoprotein B
Apo CIII	apolipoprotein CIII
Apo ER2	apolipoprotein E receptor 2
BMI	body mass index
CRPC	castration-resistant prostate cancer
FPSA	free prostate specific antigen
GLM	generalized linear model
HDL	high-density lipoprotein cholesterol
LDLR	LDL receptor
LRP1	LDLR-related protein 1
LPL	lipoprotein lipase
Lp(a)	lipoprotein(a)
lPCa	localized prostate cancer
LDL	low-density lipoprotein cholesterol
LNCaP	lymph node carcinoma of the prostate
MHC-1	major histocompatibility complex 1
mPCa	metastatic prostate cancer
non-HDL	non-HDL cholesterol
PCSK9	proprotein convertase subtilisin/kexin type 9
PCa	prostate cancer
PSA	prostate specific antigen
TC	total cholesterol
TPSA	total prostate specific antigen
TRL	triglyceride-rich lipoprotein
Trig	triglycerides
VLDL	very-low-density lipoprotein

## Appendix A

**Table A1:** Multiple Regression analysis of triglycerides and hyperlipidemic factors. Significant pairwise associations found between triglycerides and hyperlipidemic factors using partial Spearman correlations in the entire cohort (*N* = 105) and each subgroup (*n* = 35) were further investigated to determine which proteins most tightly regulated triglyceride levels. Triglycerides were significantly associated with total cholesterol, LDL, non-HDL, Apo CIII and leptin. Total cholesterol, LDL, and non-HDL were highly collinear and were removed from the analysis before a multiple regression was performed with age and BMI as covariates. Significance thresholds were defined as follows: *** *p* < 0.001, **** *p* < 0.0001.

	Estimate std.	Error	t Value	Pr (>|t|)	
Entire cohort					
(Intercept)	0.202	0.919	0.220	0.826	
Apo CIII	0.00885	0.00113	7.836	5.12 × 10^−12^	****
Leptin	0.0101	0.00691	1.462	0.147	
Age	0.000943	0.00990	0.095	0.924	
BMI	0.0108	0.0217	0.499	0.619	
At risk					
(Intercept)	0.536	1.927	0.278	0.783	
Apo CIII	0.01463	0.00281	5.207	0.000013	****
Leptin	0.0000644	0.0207	0.003	0.998	
Age	−0.0329	0.0214	−1.54	0.134	
BMI	0.0610	0.0497	1.228	0.229	
Localized					
(Intercept)	1.581	1.005	1.574	0.126	
Apo CIII	0.00611	0.00135	4.518	0.0000906	***
Leptin	0.0485	0.0112	4.309	0.000162	***
Age	−0.0126	0.0111	−1.128	0.268	
BMI	−0.0129	0.0205	−0.628	0.535	
Metastatic					
(Intercept)	0.768	1.727	0.455	0.660	
Apo CIII	0.00727	0.00181	4.016	0.000366	***
Leptin	0.00612	0.0100	0.611	0.546	
Age	0.00260	0.0192	0.135	0.893	
BMI	0.00315	0.0417	0.075	0.940	

**Table A2:** Age, BMI, ECOG and localization of metastases in the metastatic cohort.

	Mean (SD)	Median	[Min, Max]
Age	73.4 (8.7)	75	[58, 90]
BMI	27.54 (4.08)	26.7	[20.10, 39.00]
ECOG at prostatectomy	0 (*n* = 33)		
	1 (*n* = 2)		
Localization of metastases	Bone only: 23		
	Bone and ganglion: 2		
	Bone and liver: 3		
	Bone and bladder: 2		
	Bone and prostate: 1		
	Ganglion and lung: 1		
	Ganglion and progression: 3		

## References

[1] Brenner D.R., Gillis J., Demers A.A., Ellison L.F., Billette J.-M., Zhang S.X., Liu J.L., Woods R.R., Finley C., Fitzgerald N. (2024). Projected estimates of cancer in Canada in 2024. Can. Med. Assoc. J..

[2] Hamdy F.C., Donovan J.L., Lane J.A., Metcalfe C., Davis M., Turner E.L., Martin R.M., Young G.J., Walsh E.I., Bryant R.J. (2023). Fifteen-Year Outcomes after Monitoring, Surgery, or Radiotherapy for Prostate Cancer. N. Engl. J. Med..

[3] Simon N.I., Parker C., Hope T.A., Paller C.J. (2022). Best Approaches and Updates for Prostate Cancer Biochemical Recurrence. Am. Soc. Clin. Oncol. Educ. Book..

[4] Kirby M., Hirst C., Crawford E.D. (2011). Characterising the castration-resistant prostate cancer population: A systematic review. Int. J. Clin. Pract..

[5] Abdelwahed K.S., Siddique A.B., Ebrahim H.Y., Qusa M.H., Mudhish E.A., Rad A.H., Zerfaoui M., Abd Elmageed Z.Y., El Sayed K.A. (2023). Pseurotin A Validation as a Metastatic Castration-Resistant Prostate Cancer Recurrence-Suppressing Lead via PCSK9-LDLR Axis Modulation. Marine Drugs.

[6] Murtola T.J., Kasurinen T.V.J., Talala K., Taari K., Tammela T.L.J., Auvinen A. (2019). Serum cholesterol and prostate cancer risk in the Finnish randomized study of screening for prostate cancer. Prostate Cancer Prostatic Dis..

[7] Jamnagerwalla J., Howard L.E., Allott E.H., Vidal A.C., Moreira D.M., Castro-Santamaria R., Andriole G.L., Freeman M.R., Freedland S.J. (2018). Serum cholesterol and risk of high-grade prostate cancer: Results from the REDUCE study. Prostate Cancer Prostatic Dis..

[8] Hernández-Pérez J.G., Torres-Sánchez L., Hernández-Alcaráz C., López-Carrillo L., Rodríguez-Covarrubias F., Vázquez-Salas R.A., Galván-Portillo M. (2022). Metabolic Syndrome and Prostate Cancer Risk: A Population Case-control Study. Arch. Med. Res..

[9] Lee G., Han K., Lee S.-S. (2022). Different effect of obesity and metabolic syndrome on prostate cancer by age group. Am. J. Cancer Res..

[10] Koyama T., Ogawara K., Kasamatsu A., Okamoto A., Kasama H., Minakawa Y., Shimada K., Yokoe H., Shiiba M., Tanzawa H. (2015). ANGPTL3 is a novel biomarker as it activates ERK/MAPK pathway in oral cancer. Cancer Med..

[11] Zhao T., Liang X., Chen J., Bao Y., Wang A., Gan X., Lu X., Wang L. (2019). ANGPTL3 inhibits renal cell carcinoma metastasis by inhibiting VASP phosphorylation. Biochem. Biophys. Res. Commun..

[12] Zhang Y.J., Zhang L., Feng F., Cao Q.F. (2021). ANGPTL3 Overexpression Suppresses the Development of Oncogenic Properties in Renal Cell Carcinoma via the Wnt/β-Catenin Signaling Pathway and Predicts Good Prognosis. Dis. Markers.

[13] Wong Chong E., Joncas F.-H., Douville P., Bachvarov D., Diorio C., Calon F., Bergeron A.-C., Blais J., Leung S.O.A., Seidah N.G. (2024). Pre-operative levels of angiopoietin protein-like 3 (ANGPTL3) in women diagnosed with high-grade serous carcinoma of the ovary. Lipids Health Dis..

[14] Wu Y., Zheng Y., Jin Z. (2023). ANGPTL3 affects the metastatic potential and the susceptibility of ovarian cancer cells to natural killer cell-mediated cytotoxicity. Heliyon.

[15] Ishikawa M., Kitayama J., Nagawa H. (2004). Enhanced Expression of Leptin and Leptin Receptor (OB-R) in Human Breast Cancer. Clin. Cancer Res..

[16] Alshaker H., Sacco K., Alfraidi A., Muhammad A., Winkler M., Pchejetski D. (2015). Leptin signalling, obesity and prostate cancer: Molecular and clinical perspective on the old dilemma. Oncotarget.

[17] Fan Y., Gan Y., Shen Y., Cai X., Song Y., Zhao F., Yao M., Gu J., Tu H. (2015). Leptin signaling enhances cell invasion and promotes the metastasis of human pancreatic cancer via increasing MMP-13 production. Oncotarget.

[18] Saglam K., Aydur E., Yilmaz M., Göktaş S. (2003). Leptin influences cellular differentiation and progression in prostate cancer. J. Urol..

[19] Chang S., Hursting S.D., Contois J.H., Strom S.S., Yamamura Y., Babaian R.J., Troncoso P., Scardino P.T., Wheeler T.M., Amos C.I. (2001). Leptin and prostate cancer. The Prostate.

[20] Philp L.K., Rockstroh A., Sadowski M.C., Taherian Fard A., Lehman M., Tevz G., Libério M.S., Bidgood C.L., Gunter J.H., McPherson S. (2021). Leptin antagonism inhibits prostate cancer xenograft growth and progression. Endocr. Relat. Cancer.

[21] Kashiwagi E., Kawahara T., Kinoshita F., Shiota M., Inokuchi J., Miyamoto H., Eto M. (2024). The Role of Adipocytokines and their Receptors in Prostate Cancer: Adiponectin May Protect Against Progression. Anticancer. Res..

[22] Wong Chong E., Joncas F.-H., Seidah N.G., Calon F., Diorio C., Gangloff A. (2022). Circulating levels of PCSK9, ANGPTL3 and Lp(a) in stage III breast cancers. BMC Cancer.

[23] Rademaker G., Hernandez G.A., Seo Y., Dahal S., Miller-Phillips L., Li A.L., Peng X.L., Luan C., Qiu L., Liegeois M.A. (2025). PCSK9 drives sterol-dependent metastatic organ choice in pancreatic cancer. Nature.

[24] Nejabat M., Hadizadeh F., Almahmeed W., Sahebkar A. (2025). Effects of PCSK9 inhibitors on cancer, diabetes, and cardiovascular diseases. Drug Discov. Today.

[25] Cui C., Yan A., Huang S., Chen Y., Zhao J., Li C., Wang X., Yang J. (2025). PCSK9 Manipulates Lipid Metabolism and the Immune Microenvironment in Cancer. OncoTargets Ther..

[26] Ioannidou A., Watts E.L., Perez-Cornago A., Platz E.A., Mills I.G., Key T.J., Travis R.C., Tsilidis K.K., Zuber V. (2022). The relationship between lipoprotein A and other lipids with prostate cancer risk: A multivariable Mendelian randomisation study. PLoS Med..

[27] Marrer É., Wagner A., Montaye M., Luc G., Amouyel P., Dallongeville J., Ducimetiere P., Bingham A., Arveiler D., Velten M. (2013). Lipoprotein(a) plasma levels and the risk of cancer: The PRIME study. Eur. J. Cancer Prev..

[28] Wang F.M., Zhang Y. (2019). High Lipoprotein(a) Level Is Independently Associated with Adverse Clinicopathological Features in Patients with Prostate Cancer. Dis. Markers.

[29] Sylvers-Davie K.L., Davies B.S.J. (2021). Regulation of lipoprotein metabolism by ANGPTL3, ANGPTL4, and ANGPTL8. Am. J. Physiol.-Endocrinol. Metab..

[30] Chen P.Y., Gao W.Y., Liou J.W., Lin C.Y., Wu M.J., Yen J.H. (2021). Angiopoietin-Like Protein 3 (ANGPTL3) Modulates Lipoprotein Metabolism and Dyslipidemia. Int. J. Mol. Sci..

[31] Nordestgaard A.T., Tybjærg-Hansen A., Mansbach H., Kersten S., Nordestgaard B.G., Rosenson R.S. (2025). Target Populations for Novel Triglyceride-Lowering Therapies. J. Am. Coll. Cardiol..

[32] Borén J., Packard C.J., Taskinen M.-R. (2020). The Roles of ApoC-III on the Metabolism of Triglyceride-Rich Lipoproteins in Humans. Front. Endocrinol..

[33] Gordts P.L., Nock R., Son N.H., Ramms B., Lew I., Gonzales J.C., Thacker B.E., Basu D., Lee R.G., Mullick A.E. (2016). ApoC-III inhibits clearance of triglyceride-rich lipoproteins through LDL family receptors. J. Clin. Invest..

[34] Giammanco A., Spina R., Cefalù A.B., Averna M. (2023). APOC-III: A Gatekeeper in Controlling Triglyceride Metabolism. Curr. Atheroscler. Rep..

[35] Maffei M., Halaas J., Ravussin E., Pratley R.E., Lee G.H., Zhang Y., Fei H., Kim S., Lallone R., Ranganathan S. (1995). Leptin levels in human and rodent: Measurement of plasma leptin and ob RNA in obese and weight-reduced subjects. Nat. Med..

[36] Friedman J.M., Halaas J.L. (1998). Leptin and the regulation of body weight in mammals. Nature.

[37] Zeng W., Pirzgalska R.M., Pereira M.M.A., Kubasova N., Barateiro A., Seixas E., Lu Y.-H., Kozlova A., Voss H., Martins G.G. (2015). Sympathetic Neuro-adipose Connections Mediate Leptin-Driven Lipolysis. Cell.

[38] Picó C., Palou M., Pomar C.A., Rodríguez A.M., Palou A. (2022). Leptin as a key regulator of the adipose organ. Rev. Endocr. Metab. Disord..

[39] Hackl M.T., Fürnsinn C., Schuh C.M., Krssak M., Carli F., Guerra S., Freudenthaler A., Baumgartner-Parzer S., Helbich T.H., Luger A. (2019). Brain leptin reduces liver lipids by increasing hepatic triglyceride secretion and lowering lipogenesis. Nat. Commun..

[40] Beghini M., Metz M., Baumgartner C., Wolf P., Bastian M., Hackl M., Baumgartner-Parzer S., Marculescu R., Krebs M., Harreiter J. (2025). Leptin acutely increases hepatic triglyceride secretion in patients with lipodystrophy. Metabolism.

[41] Schörghofer D., Kinslechner K., Preitschopf A., Schütz B., Röhrl C., Hengstschläger M., Stangl H., Mikula M. (2015). The HDL receptor SR-BI is associated with human prostate cancer progression and plays a possible role in establishing androgen independence. Reprod. Biol. Endocrinol..

[42] Raftopulos N.L., Washaya T.C., Niederprüm A., Egert A., Hakeem-Sanni M.F., Varney B., Aishah A., Georgieva M.L., Olsson E., Dos Santos D.Z. (2022). Prostate cancer cell proliferation is influenced by LDL-cholesterol availability and cholesteryl ester turnover. Cancer Metab..

[43] Jin H.-R., Wang J., Wang Z.-J., Xi M.-J., Xia B.-H., Deng K., Yang J.-L. (2023). Lipid metabolic reprogramming in tumor microenvironment: From mechanisms to therapeutics. J. Hematol. Oncol..

[44] Corn K.C., Windham M.A., Rafat M. (2020). Lipids in the tumor microenvironment: From cancer progression to treatment. Prog. Lipid Res..

[45] Feingold K.R., Grunfeld C., Johnstone M., Veves A. (2023). Diabetes and Dyslipidemia. Diabetes and Cardiovascular Disease.

[46] Siltari A., Syvälä H., Lou Y.R., Gao Y., Murtola T.J. (2022). Role of Lipids and Lipid Metabolism in Prostate Cancer Progression and the Tumor’s Immune Environment. Cancers.

[47] Tousignant K.D., Rockstroh A., Taherian Fard A., Lehman M.L., Wang C., McPherson S.J., Philp L.K., Bartonicek N., Dinger M.E., Nelson C.C. (2019). Lipid Uptake Is an Androgen-Enhanced Lipid Supply Pathway Associated with Prostate Cancer Disease Progression and Bone Metastasis. Mol. Cancer Res..

[48] Suh J., Shin T.J., You D., Jeong I.G., Hong J.H., Kim C.-S., Ahn H. (2023). The association between serum lipid profile and the prostate cancer risk and aggressiveness. Front. Oncol..

[49] Allott E.H., Howard L.E., Cooperberg M.R., Kane C.J., Aronson W.J., Terris M.K., Amling C.L., Freedland S.J. (2014). Serum lipid profile and risk of prostate cancer recurrence: Results from the SEARCH database. Cancer Epidemiol. Biomarkers Prev..

[50] Kersten S. (2021). Role and mechanism of the action of angiopoietin-like protein ANGPTL4 in plasma lipid metabolism. J. Lipid Res..

[51] Zhong L., Tang L., He X. (2022). Angiopoietin-like 3 (ANGPTL3) drives cell proliferation, migration and angiogenesis in cervical cancer via binding to integrin alpha v beta 3. Bioengineered.

[52] Wang J., Man Q., Zhong N., Wang H., Zhang C., Li S., Bu L., Liu B. (2022). ENO1 Binds to ApoC3 and Impairs the Proliferation of T Cells via IL-8/STAT3 Pathway in OSCC. Int. J. Mol. Sci..

[53] Haynes W.G., Morgan D.A., Walsh S.A., Mark A.L., Sivitz W.I. (1997). Receptor-mediated regional sympathetic nerve activation by leptin. J. Clin. Invest..

[54] Modak S., Aktar T., Majumder D., Singha A.K., Maiti D. (2025). A systemic review on leptin’s role in defining cancer: Special emphasis on immunomodulation, inflammation, and therapeutic interventions. Genes. Immun..

[55] Liu X., Bao X., Hu M., Chang H., Jiao M., Cheng J., Xie L., Huang Q., Li F., Li C.Y. (2020). Inhibition of PCSK9 potentiates immune checkpoint therapy for cancer. Nature.

[56] Demers A., Samami S., Lauzier B., Des Rosiers C., Ngo Sock E.T., Ong H., Mayer G. (2015). PCSK9 Induces CD36 Degradation and Affects Long-Chain Fatty Acid Uptake and Triglyceride Metabolism in Adipocytes and in Mouse Liver. Arterioscler. Thromb. Vasc. Biol..

[57] Chen Y., Zhang J., Cui W., Silverstein R.L. (2022). CD36, a signaling receptor and fatty acid transporter that regulates immune cell metabolism and fate. J. Exp. Med..

[58] Weinstock P.H., Levak-Frank S., Hudgins L.C., Radner H., Friedman J.M., Zechner R., Breslow J.L. (1997). Lipoprotein lipase controls fatty acid entry into adipose tissue, but fat mass is preserved by endogenous synthesis in mice deficient in adipose tissue lipoprotein lipase. Proc. Natl. Acad. Sci. USA.

[59] Liu X., Zhang Y., Han B., Li L., Li Y., Ma Y., Kang S., Li Q., Kong L., Huang K. (2024). Postprandial exercise regulates tissue-specific triglyceride uptake through angiopoietin-like proteins. JCI Insight.

[60] Ham J.M., Jones M., Kemp D. (1972). Platelet adhesiveness and lipoprotein lipase activity in patients with benign and malignant disease of the prostate. Br. J. Surg..

[61] Bavis M.M., Nicholas A.M., Tobin A.J., Christian S.L., Brown R.J. (2023). The breast cancer microenvironment and lipoprotein lipase: Another negative notch for a beneficial enzyme?. FEBS Open Bio.

[62] Kuemmerle N.B., Rysman E., Lombardo P.S., Flanagan A.J., Lipe B.C., Wells W.A., Pettus J.R., Froehlich H.M., Memoli V.A., Morganelli P.M. (2011). Lipoprotein lipase links dietary fat to solid tumor cell proliferation. Mol. Cancer Ther..

[63] Bancaro N., Calì B., Troiani M., Elia A.R., Arzola R.A., Attanasio G., Lai P., Crespo M., Gurel B., Pereira R. (2023). Apolipoprotein E induces pathogenic senescent-like myeloid cells in prostate cancer. Cancer Cell.

[64] Liu R.Z., Godbout R. (2020). An Amplified Fatty Acid-Binding Protein Gene Cluster in Prostate Cancer: Emerging Roles in Lipid Metabolism and Metastasis. Cancers.

[65] Narita S., Tsuchiya N., Wang L., Matsuura S., Ohyama C., Satoh S., Sato K., Ogawa O., Habuchi T., Kato T. (2004). Association of lipoprotein lipase gene polymorphism with risk of prostate cancer in a Japanese population. Int. J. Cancer.

[66] Kim J.W., Cheng Y., Liu W., Li T., Yegnasubramanian S., Zheng S.L., Xu J., Isaacs W.B., Chang B.L. (2009). Genetic and epigenetic inactivation of LPL gene in human prostate cancer. Int. J. Cancer.

[67] Leon C.G., Locke J.A., Adomat H.H., Etinger S.L., Twiddy A.L., Neumann R.D., Nelson C.C., Guns E.S., Wasan K.M. (2010). Alterations in cholesterol regulation contribute to the production of intratumoral androgens during progression to castration-resistant prostate cancer in a mouse xenograft model. The Prostate.

[68] Seidah N.G., Prat A. (2021). The Multifaceted Biology of PCSK9. Endocr. Rev..

[69] Ajoolabady A., Pratico D., Mazidi M., Davies I.G., Lip G.Y.H., Seidah N., Libby P., Kroemer G., Ren J. (2025). PCSK9 in metabolism and diseases. Metabolism.

[70] Mahboobnia K., Pirro M., Marini E., Grignani F., Bezsonov E.E., Jamialahmadi T., Sahebkar A. (2021). PCSK9 and cancer: Rethinking the link. Biomed. Pharmacother..

[71] Ben Djoudi Ouadda A., Gauthier M.S., Susan-Resiga D., Girard E., Essalmani R., Black M., Marcinkiewicz J., Forget D., Hamelin J., Evagelidis A. (2019). Ser-Phosphorylation of PCSK9 (Proprotein Convertase Subtilisin-Kexin 9) by Fam20C (Family With Sequence Similarity 20, Member C) Kinase Enhances Its Ability to Degrade the LDLR (Low-Density Lipoprotein Receptor). Arterioscler. Thromb. Vasc. Biol..

[72] Hentze H., Jensen K.K., Chia S.M., Johns D.G., Shaw R.J., Davis H.R., Shih S.-J., Wong K.K. (2013). Inverse relationship between LDL cholesterol and PCSK9 plasma levels in dyslipidemic cynomolgus monkeys: Effects of LDL lowering by ezetimibe in the absence of statins. Atherosclerosis.

[73] Mayne J., Dewpura T., Raymond A., Cousins M., Chaplin A., Lahey K.A., LaHaye S.A., Mbikay M., Ooi T.C., Chrétien M. (2008). Plasma PCSK9 levels are significantly modified by statins and fibrates in humans. Lipids Health Dis..

[74] Zhang B., Chuang G.Y., Biju A., Biner D., Cheng J., Wang Y., Bao S., Chao C.W., Lei H., Liu T. (2024). Cholesterol reduction by immunization with a PCSK9 mimic. Cell Rep..

[75] Ference B.A., Robinson J.G., Brook R.D., Catapano A.L., Chapman M.J., Neff D.R., Voros S., Giugliano R.P., Davey Smith G., Fazio S. (2016). Variation in PCSK9 and HMGCR and Risk of Cardiovascular Disease and Diabetes. N. Engl. J. Med..

[76] Cohen J.C., Boerwinkle E., Mosley T.H., Hobbs H.H. (2006). Sequence variations in PCSK9, low LDL, and protection against coronary heart disease. N. Engl. J. Med..

[77] Marques R.B., Dits N.F., Erkens-Schulze S., van Weerden W.M., Jenster G. (2010). Bypass Mechanisms of the Androgen Receptor Pathway in Therapy-Resistant Prostate Cancer Cell Models. PLoS ONE.

[78] Faris J.E., Smith M.R. (2010). Metabolic consequences associated with androgen deprivation therapy for prostate cancer. Curr. Opin. Endocrinol. Diabetes Obes..

[79] Smith M.R., Finkelstein J.S., McGovern F.J., Zietman A.L., Fallon M.A., Schoenfeld D.A., Kantoff P.W. (2002). Changes in body composition during androgen deprivation therapy for prostate cancer. J. Clin. Endocrinol. Metab..

[80] Dockery F., Bulpitt C.J., Agarwal S., Donaldson M., Rajkumar C. (2003). Testosterone suppression in men with prostate cancer leads to an increase in arterial stiffness and hyperinsulinaemia. Clin. Sci..

[81] Eri L.M., Urdal P., Bechensteen A.G. (1995). Effects of the luteinizing hormone-releasing hormone agonist leuprolide on lipoproteins, fibrinogen and plasminogen activator inhibitor in patients with benign prostatic hyperplasia. J. Urol..

[82] Siamakpour-Reihani S., Owzar K., Jiang C., Turner T., Deng Y., Bean S.M., Horton J.K., Berchuck A., Marks J.R., Dewhirst M.W. (2015). Prognostic significance of differential expression of angiogenic genes in women with high-grade serous ovarian carcinoma. Gynecol. Oncol..

